# Determining the frequency of thyroid parameter measurements following rhTSH administration in a healthy, older population

**DOI:** 10.1016/j.mex.2021.101400

**Published:** 2021-05-28

**Authors:** Ana Zutinic, Gerard J. Blauw, Diana van Heemst

**Affiliations:** Department of Internal Medicine, Division of Gerontology and Geriatrics, Leiden University Medical Center, Leiden, the Netherlands

**Keywords:** Thyroid, Longevity, Geriatrics, Blood sampling, rhTSH

## Abstract

Serial thyroid hormone measurement in blood following recombinant human thyroid stimulating hormone (rhTSH) administration has not been studied extensively in healthy, older populations. Current methods involve measurement of thyroid hormones mostly at 4 to 24 hours following rhTSH administration. We tailored existing protocols to measure thyroid hormones at high frequencies following 0.1mg rhTSH intramuscular (i.m.) administration to identify optimal measurement points in our healthy, older population. We designed a method with frequent blood sampling in the first 8 hours, followed by blood sampling at 24, 48 and 72 hours after rhTSH administration to measure TSH, thyroxine (T4), free T4 (fT4), triiodothyronine (T3), free T3 (fT3) and thyroglobulin (Tg). In total, we performed a series of 17 blood withdrawals in four consecutive days. Following 0.1mg rhTSH (i.m.) administration, mean thyroid parameters showed great inter-individual variation and variation over time. Mean TSH concentration showed the greatest variation in the first 8 hours following rhTSH administration. Mean T4, fT4, T3 and fT3 started showing variation from 2 hours after rhTSH administration, and were less variable than mean TSH concentration. Mean Tg was only variable at later time points, namely 24, 48 and 72 hours after rhTSH administration. In this novel method with high frequency blood sampling following 0.1mg rhTSH (i.m.) administration, we identify optimal time points for measuring thyroid gland output in a healthy, older population. Our methods and findings may be informative for further thyroid but also other hormonal axis studies.

• Thyroid metabolism
• Blood sampling frequency
• Geriatrics and longevity

## Graphical abstract

**Figure:**
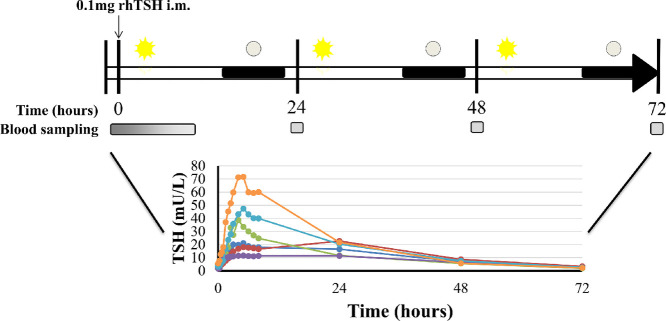

Specifications table

**Table:** 

Subject area:	Medicine and Dentistry
More specific subject area:	Endocrinology and geriatrics
Method name:	Blood sampling frequency following recombinant human thyroid stimulating hormone administration
Name and reference of original method:	N/A
Resource availability:	N/A

## Background

Thyroid stimulating hormone (TSH) is the main driver of thyroid hormone production by the thyroid gland. Before characterization of the TSH gene, bovine as well as human cadaver TSH have been isolated for use in the clinic for detecting residual disease in patients with thyroid cancer [1]. Following the characterization of the TSH subunits [2,3], recombinant human TSH (rhTSH) could be produced, in Chinese hamster ovary cells [4], and since then rhTSH has been used in the clinic as well as in preclinical studies comprising healthy young and middle-aged adult populations. Current literature provides limited information on the magnitude and variation in effects of rhTSH on the output of the thyroid gland in a healthy, older population.

In the Leiden Longevity Study (LLS), we recently showed that offspring from long-lived families have a different thyroid axis status than controls, albeit in the normal range, with offspring displaying higher circulating TSH levels than controls in the absence of differences in levels of thyroid hormones over a 24-hour period as measured every 10 minutes [5]. Based on this finding, the hypothesis was formulated that the thyroid gland in offspring of long-lived families is less responsive to TSH stimulation than the thyroid gland of controls. To test this hypothesis, we aimed to perform a thyroid axis challenge study with rhTSH in a healthy, older population.

Previous literature studying rhTSH dynamics reported effects on young and middle-aged adults, mostly measured at 24 hour intervals [6], [7], [8], although some studies reported thyroid parameter concentrations at 2 and 4 hours following rhTSH administration [9,10]. However, since there is great variation in the magnitude of changes in thyroid parameters following rhTSH administration [7,10] and since there were indications that age modifies the response to rhTSH [7,11], uncertainty remained about the appropriate frequency of blood sampling for thyroid parameter measurements in our healthy, older population.

We performed a series of frequent blood sampling and measured thyroid parameters TSH, thyroxine (T4), free T4 (fT4), triiodothyronine (T3), free T3 (fT3) and thyroglobulin (Tg) in a small group comprising 6 participants, in order to determine the necessary frequency of measurement in the whole cohort.

Here we report the method used to identify optimal time points for measurement of thyroid hormone parameters following intramuscular administration of 0.1mg rhTSH in a healthy, older population.

## Method details

### Study population

Study participants were recruited from LLS [12], underwent a medical screening and were excluded based on criteria outlined in detail elsewhere [13]. All participants gave written informed consent and the study was performed in accordance with the declaration of Helsinki.

### Blood sampling frequency

On the morning of study day 1, an intravenous cannula was placed in a forearm vein, blood was withdrawn at baseline and rhTSH was administered through intramuscular injection (0.1 mg/mL in 1 mL, gluteal muscle). The time of injection was used as reference, time zero. Blood was sampled at a high frequency following injection for optimal detection of circulating parameters reflecting the thyroidal response to rhTSH. In the first hour after injection, blood was sampled every 15 min. Between 1 and 3 h after injection, blood was sampled every 30 min, and finally between 3 and 8 h after injection, every hour. During study day 1, subjects received two standardized meals (two hours and five hours after rhTSH injection), each consisting of 600 kcal (2 × 125mL Nutridrink Compact, Nutricia Advanced Medical Nutrition, Zoetermeer, The Netherlands). On study day 2, 3 and 4, additional fasted blood samples were obtained at respectively 24, 48 and 72 h after rhTSH injection.

This is the highest frequency of sampling reported in literature following a rhTSH challenge.

### Serum samples and laboratory measurements

Serum samples were kept at room temperature for 60 min to clot before processing at the Department of Clinical Chemistry and Laboratory Medicine, Leiden University Medical Centre, The Netherlands. Samples were centrifuged for 10 min at 2350 G relative centrifugal force at a temperature of 20 degrees Celsius. After being transferred to 500 microliter aliquots, serum samples were stored at –20 degrees Celsius prior to permanent storage at –80 degrees Celsius until analysis.

Laboratory measurements were performed after all subjects had completed the study. All measurements were performed with the same lot number. For each participant, samples from the different time points were measured in the same batch. Assays and assay performance are reported in detail elsewhere [13]. In short, all measurements were performed with fully automated, software monitored equipment and diagnostics from Roche Diagnostics (Almere, The Netherlands) at the Department of Clinical Chemistry and Laboratory Medicine at Leiden University Medical Centre, The Netherlands. Thyroid parameters TSH (Catalogue number 11731459122, research reference identifier (RRID): AB_2756377), fT4 (Catalogue number 6437281190, RRID: AB_2801661), T4 (Catalogue number 12017709122, RRID: AB_2756378), fT3 (Catalogue number 6437206190, RRID: AB_2827368) and T3 (Catalogue number 11731360122, RRID: AB_2827369) were measured in serum by an immunoassay using Roche cobas8000 with an E602 module.

### Determining thyroid parameter measurement frequency for whole cohort

Following completion of the study by all participants, samples from three female and three male (total n=6) participants who participated in both the rhTSH study as well as in a second study with T3, as a couple and for whom blood collection was successful at all time points, were used to measure thyroid parameters at all 17 time points in order to identify optimal measurement points for the whole cohort comprising 30 participants.

## Results

The baseline characteristics of the pilot study participants are shown in Table 1, showing that study participants were of high-middle age and there was an equal distribution of men and women.

**Table 1:** Baseline characteristics of the study participants (n=6).

	All participants	Males	Females
Number	6	3	3
Age *years*	68 (2)	69 (1)	67 (2)
Weight *kg*	79.2 (19.9)	85.9 (12.8)	72.6 (26.4)
Height *cm*	170.5 (10.2)	178.8 (5.55)	162.1 (4.6)
BMI *kg/m^2^*	27.1 (5.8)	26.9 (3.6)	27.2 (8.5)
GFR *ml/min/1.73m^2^*	72.8 (9.8)	70.3 (9.1)	75.3 (11.7)
AST *U/L*	20.2 (5.1)	20.1 (6.1)	20.2 (5.4)
ALT *U/L*	19.1 (7.2)	23.7 (6.4)	14.5 (5.2)

All values are shown as mean (standard deviation). BMI: body mass index; GFR: glomerular filtration rate; AST: aspartate transaminase; ALT: alanine transaminase.

Thyroid parameters at baseline and following 0.1 mg rhTSH (i.m.) administration are shown in Table 2. Participants were euthyroid at baseline. Following 0.1 mg rhTSH administration, peak values of thyroid parameters were reached, although at different time points depending on the parameter. Circulating TSH, T3 and fT3 reached peak concentrations before 24 hours (namely at 5, 7.5 and 7h, respectively) following rhTSH administration. Peak circulating fT4 and T4 was reached at 48 h following rhTSH administration, while Tg concentration peak was reached at 72 hours following rhTSH administration. The greatest increase in concentration from baseline following rhTSH administration was seen in circulating TSH, followed by circulating Tg (1013% and 217%, respectively).

**Table 2:** Thyroid parameters at baseline and following 0.1 mg i.m. rhTSH administration in all pilot study participants (n=6) and in the whole cohort (n=30).

	Baseline	Peak	% increase from baseline to peak	Time of peak (hours)	Time of peak whole cohort * (hours)
TSH *mU/L*	2.9 (1.7-5.6)	30.7 (11.5-71.2)	1013 (331-1414)	5 (4-24)	6 (3-24)
fT4 *pmol/L*	15.3 (12.2-20.6)	20.9 (17.4-45.2)	42 (31-118)	24 (24-48)	24 (8-48)
T4 *mmol/L*	85.5 (84.7-110.6)	113.3 (100.2-183.1)	32 (26-65)	24 (24-48)	24 (24-48)
fT3 *pmol/L*	4.9 (4.4-5.3)	8.5 (7.1-14.5)	69 (59-174)	7 (5-24)	24 (5-48)
T3 *mmol/L*	1.6 (1.3-1.8)	2.7 (2.1-3.6)	63 (50-110)	7.5 (4-24)	24 (4-48)
Tg *µg/L*	11.6 (3.2-56.3)	46.6 (8.3-194.9)	217 (142-465)	48 (24-72)	48 (24-72)

All values are shown as median (minimum - maximum). rhTSH: recombinant human thyroid stimulating hormone; i.m.: intramuscular. TSH: thyroid stimulating hormone; fT4: free T4; T4: thyroxine; fT3: free T3; T3: triiodothyronine; Tg: thyroglobulin. *analysis based on n=29 due to n=1 excluded from analysis due to suspected intravenous rhTSH administration.

The peak times and intervals of the whole cohort (n=30) are also presented in Table 2 for comparison with our study (n=6). In the whole cohort, the peak times and intervals of TSH, T4, fT4 and Tg were comparable to our study population, further strengthening our results. T3 and fT3 in the whole cohort had comparable minimum but slightly later peak and maximum times compared to our study, further supporting the necessity of multiple measurement time points due to large variation in a healthy, older population.

**Fig. 1:**
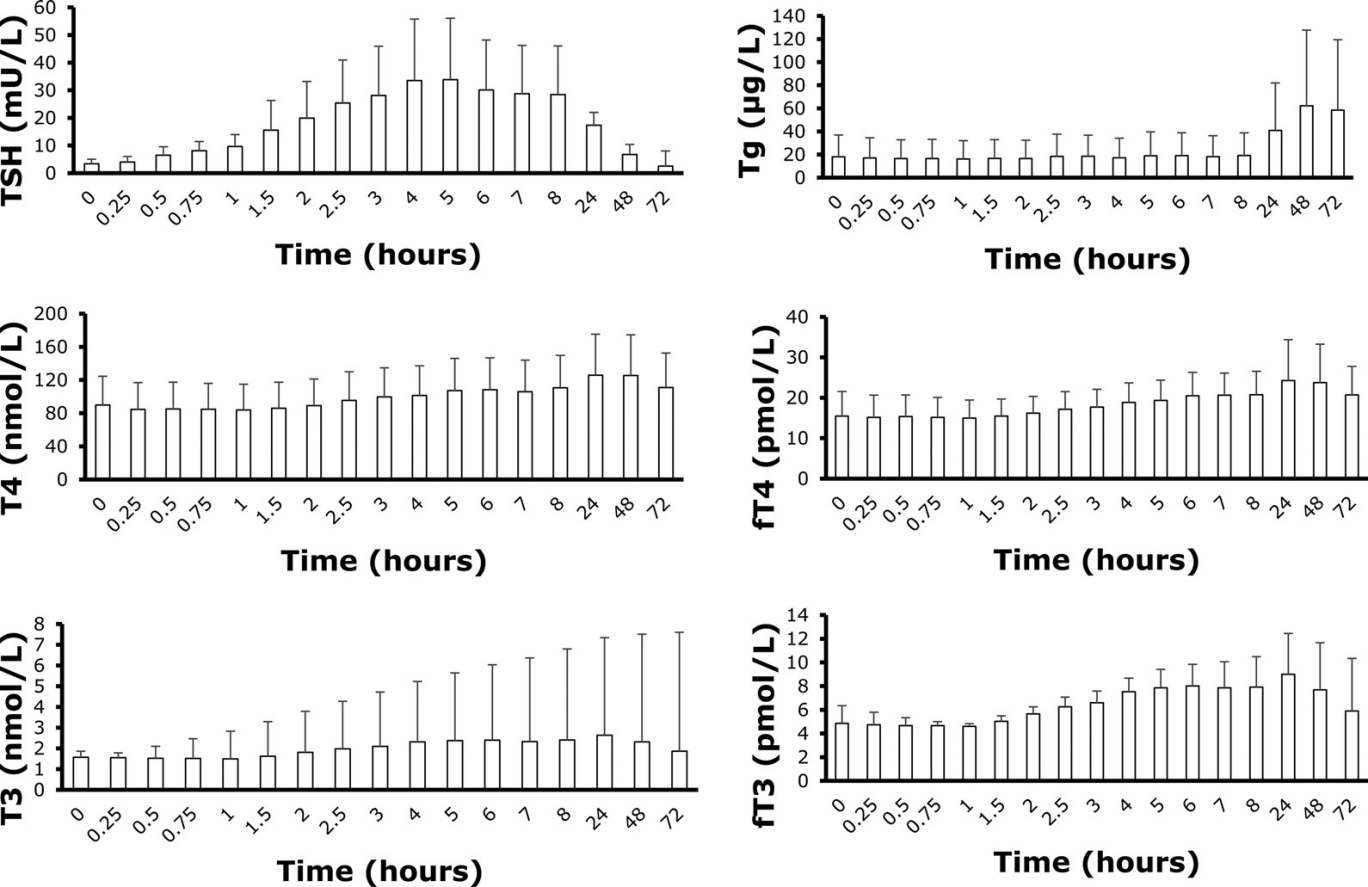
Mean thyroid parameters at baseline and at 16 timepoints divided across four consecutive days following 0.1mg rhTSH (i.m.) administration in a healthy, older population (n=6). Error bars represent standard deviation. rhTSH: recombinant human thyroid stimulating hormone; i.m.: intramuscular.

Mean (SD) thyroid parameters at baseline and following 0.1 mg rhTSH (i.m.) administration are shown in Fig. 1. Eye-level evidence suggests that during the first 8 hours following rhTSH administration, circulating TSH concentration was the most variable while Tg concentration had very low variability. In fact, circulating TSH displayed the greatest variation from baseline during the first 8 hours following administration, and values started returning to baseline 24, 48 and 72 hours following rhTSH administration. FT4 and T4 had similar variability during the first 8 hours following rhTSH administration, as did fT3 and T3.

**Fig. 2:**
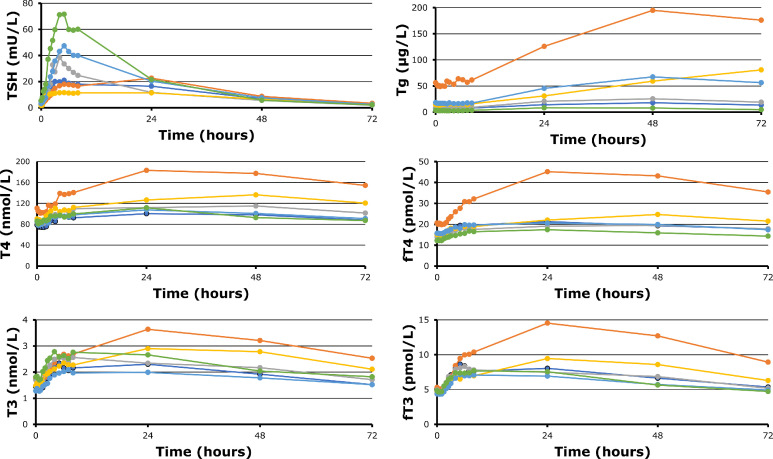
Thyroid parameters per participant in a healthy, older population (n=6) at baseline and throughout the study at 16 timepoints following 0.1mg rhTSH (i.m.) administration. rhTSH: recombinant human thyroid stimulating hormone; i.m.: intramuscular.

Thyroid parameters per participant (n=6) at baseline and following 0.1 mg rhTSH (i.m.) administration are shown in Fig. 2. Eye evidence suggests considerable inter-individual variation in circulating thyroid parameters following rhTSH administration.

Considering the inter-individual variation but also great variation over time of thyroid parameters following administration of 0.1mg rhTSH, we recommend frequent measurement of TSH (every 30-60 minutes), followed by slightly less frequent measurements of fT4, fT3, T3 and T4 (every 1-2 hours) during the first 8 hours following administration. Tg displayed very low responses during the first 8 hours following administration and may be measured at 24 hour intervals following administration.

## Conclusion

In this novel study of thyroid parameter measurement frequency following 0.1mg rhTSH (i.m.) administration in a healthy, older population, we show detailed profiles of thyroid parameters and identify optimal measurement points for recording thyroid hormone responses to rhTSH administration. Our recommendation is to, following 0.1 mg rhTSH (i.m.) administration, measure TSH concentrations at a maximum frequency of every 15 minutes in the first 8 hours and to measure fT4, T4, fT3 and T3 concentrations (or alternatively, fT4 and fT3 concentrations only, since their trajectories closely resembled those of T4 and T3) at a frequency of 2 hours in the 8 hours following administration, followed by measurements at 24, 48 and 72 hours following administration. Tg displayed very low variability in the first 8 hours, and can therefore be measured at baseline, 24, 48 and 72 hours, following rhTSH administration. In future research, these findings can be used to optimally estimate frequency of blood withdrawals and optimize protocols for blood sampling frequencies in thyroid hormone and other hormone studies, especially in older populations where literature remains scarce.

## Funding

10.13039/501100000780European Commission (10.13039/100010661Horizon 2020 research and innovation programme, 666869).

## References

[1] Nielsen V.E., Bonnema S.J., Hegedus L. (2004). The effects of recombinant human thyrotropin, in normal subjects and patients with goitre. Clin. Endocrinol. (Oxf).

[2] Wondisford F.E., Radovick S., Moates J.M., Usala S.J., Weintraub B.D. (1988). Isolation and characterization of the human thyrotropin beta-subunit gene. Differences in gene structure and promoter function from murine species. J. Biol. Chem..

[3] Hayashizaki Y., Miyai K., Kato K., Matsubara K. (1985). Molecular cloning of the human thyrotropin-beta subunit gene. FEBS Lett..

[4] Cole E.S., Lee K., Lauziere K., Kelton C., Chappel S., Weintraub B., Ferrara D., Peterson P., Bernasconi R., Edmunds T. (1993). Recombinant human thyroid stimulating hormone: development of a biotechnology product for detection of metastatic lesions of thyroid carcinoma. Biotechnology (N. Y.).

[5] Jansen S.W., Akintola A.A., Roelfsema F., van der Spoel E., Cobbaert C.M., Ballieux B.E., Egri P., Kvarta-Papp Z., Gereben B., Fekete C., Slagboom P.E., van der Grond J., Demeneix B.A., Pijl H., Westendorp R.G., van Heemst D. (2015). Human longevity is characterised by high thyroid stimulating hormone secretion without altered energy metabolism. Sci. Rep..

[6] Fast S., Nielsen V.E., Bonnema S.J., Hegedus L. (2010). Dose-dependent acute effects of recombinant human TSH (rhTSH) on thyroid size and function: comparison of 0.1, 0.3 and 0.9 mg of rhTSH. Clin. Endocrinol. (Oxf).

[7] Over R., Nsouli-Maktabi H., Burman K.D., Jonklaas J. (2010). Age modifies the response to recombinant human thyrotropin. Thyroid.

[8] Pena S., Arum S., Cross M., Magnani B., Pearce E.N., Oates M.E., Braverman L.E. (2006). 123I thyroid uptake and thyroid size at 24, 48, and 72 hours after the administration of recombinant human thyroid-stimulating hormone to normal volunteers. J. Clin. Endocrinol. Metab..

[9] Nielsen V.E., Bonnema S.J., Hegedus L. (2004). Effects of 0.9 mg recombinant human thyrotropin on thyroid size and function in normal subjects: a randomized, double-blind, cross-over trial. J. Clin. Endocrinol. Metab..

[10] Torres M.S., Ramirez L., Simkin P.H., Braverman L.E., Emerson C.H. (2001). Effect of various doses of recombinant human thyrotropin on the thyroid radioactive iodine uptake and serum levels of thyroid hormones and thyroglobulin in normal subjects. J. Clin. Endocrinol. Metab..

[11] Hautzel H., Pisar E., Lindner D., Schott M., Grandt R., Muller H.W. (2013). Impact of renal function and demographic/anthropomorphic variables on peak thyrotropin after recombinant human thyrotropin stimulation: a stepwise forward multiple-regression analysis. Thyroid.

[12] Schoenmaker M., de Craen A.J., de Meijer P.H., Beekman M., Blauw G.J., Slagboom P.E., Westendorp R.G. (2006). Evidence of genetic enrichment for exceptional survival using a family approach: the Leiden longevity study. Eur. J. Hum. Genet..

[13] Zutinic A., Pijl H., Ballieux B.E., Roelfsema F., Westendorp R.G.J., Blauw G.J., van Heemst D. (2020). Familial longevity is associated with an attenuated thyroidal response to recombinant human thyroid stimulating hormone. J. Clin. Endocrinol. Metab..

